# T_1_‐Dark Rim as a Marker of New and Chronic Active Multiple Sclerosis Lesions: A Serial Study With Frequent 7T MRI

**DOI:** 10.1111/jon.70044

**Published:** 2025-05-13

**Authors:** Madeleine Marshall, Kingkarn Aphiwatthanasumet, Olivier Mougin, Christine Stadelmann, Paul S. Morgan, Rob A. Dineen, Penny Gowland, Nikos Evangelou, Margareta A. Clarke

**Affiliations:** ^1^ Mental Health and Clinical Neurosciences Academic Unit, School of Medicine University of Nottingham Nottingham UK; ^2^ Sir Peter Mansfield Imaging Centre, School of Physics and Astronomy University of Nottingham Nottingham UK; ^3^ Department of Radiological Technology Allied Health Sciences, Naresuan University Phitsanulok Thailand; ^4^ Department of Neuropathology University Medical Centre Göttingen Göttingen Germany; ^5^ NIHR Nottingham Biomedical Research Centre Nottingham UK

**Keywords:** 7T, chronic active lesion, MP2RAGE, multiple sclerosis, paramagnetic rim lesion, quantitative susceptibility mapping, T_1_‐dark rim

## Abstract

**Background and Purpose:**

Chronic active multiple sclerosis (MS) lesions represent a particularly destructive subset of lesions on pathology. However, their imaging correlates, including paramagnetic rim lesions (PRLs) detected on susceptibility‐weighted imaging, lack sensitivity and are difficult to implement in clinical practice. This exploratory, longitudinal study investigates the prevalence and temporal dynamics of a novel imaging marker, T_1_‐dark rims, and their relationship with PRLs observed on quantitative susceptibility mapping (QSM).

**Methods:**

Four untreated people with MS underwent 7‐Tesla MRI scanning six times over a period of 36 weeks. New and pre‐existing lesions were analyzed for the presence and temporal evolution of T_1_‐dark and QSM rims. Quantitative T_1_ values were derived using B_1_ maps, and the relationship between rim status and lesion size was evaluated.

**Results:**

Of the 159 baseline lesions, 22 (14%) had T_1_‐dark rims, 11 (7%) had QSM rims, and five lesions had both. T_1_‐dark and QSM rims showed temporal changes, with T_1_‐dark rims preceding new QSM rim appearance in three out of four (75%) lesions. Eleven out of 20 (55%) newly formed lesions had T_1_‐dark rims, with a T_1_‐dark rim present in all new lesions over 100 mm^3^. Small new lesions lacked discernible rims, but their overall T_1_ values aligned with those of larger lesion T_1_‐dark rims implying shared pathological processes.

**Conclusions:**

T_1_‐dark rims were more common than QSM rims, with greater prevalence in newly formed lesions. We propose they represent edema and inflammation associated with early stages of chronic active lesion formation visible despite, not because of, iron accumulation.

## 1 Introduction

Multiple sclerosis (MS) is an autoimmune disease characterized by the presence of demyelinating lesions in the CNS. Postmortem studies of acute MS lesions have revealed that oligodendrocyte apoptosis, immune cell influx, and inflammation occur at the initial stages of lesion formation around a central vein, accompanied by the opening of the blood–brain barrier (BBB) [1, 2]. Consequent fluid leakage into the perivascular space leads to edema [3], followed by destruction and phagocytosis of myelin at the center of the lesion, which may continue at the lesion edges during the chronic active stage [1].

Being able to characterize specific pathological processes associated with different stages of MS lesion evolution in vivo is one of the main goals of neuroimaging research in MS [4]. MRI is routinely used to identify new lesions and monitor the evolution of existing lesions at the time of diagnosis and throughout disease duration. With the use of different MRI sequences, significant research progress has been made toward visualizing specific pathological processes beyond merely just visualizing the presence of lesions.

Analysis of dynamic contrast enhancement has proposed two patterns of gadolinium enhancement seen in newly formed lesions. Initially, center‐out (also called centrifugal) enhancement is detected, which is thought to reflect the outward formation of a lesion from a central vein. Within days, this shifts to the periphery‐in (also called centripetal) pattern [5, 6]. This is thought to reflect the closure of the BBB around the central vein and opening of the BBB in the peripheral vasculature as the lesion expands, with a concomitant shift in inflammation from the center to the periphery. Once the BBB is closed, accompanied by the resolution of gadolinium enhancement on MRI, a proportion of lesions evolve into chronic active lesions (CALs).

CALs have persistent, active inflammation at the edge of the demyelinated lesion thought to be driven by iron‐laden macrophages and microglia [7]. As a result, some CALs can be identified on susceptibility‐weighted imaging (SWI) as paramagnetic rim lesions (PRLs), which are characterized by the presence of a hypointense or hyperintense rim (depending on the sequence used) [8]. Pathological studies report that up to 57% of lesions are chronic active [9], whereas PRLs represent around 10% of all lesions [10].

Recently, Naval‐Baudin et al. described a novel imaging sign on 3T high‐resolution turbo field echo T_1_‐weighted imaging, termed the T_1_‐dark rim [11]. The authors suggested that T_1_‐dark rims have higher sensitivity for detecting PRLs compared to SWI, including phase maps, and that T_1_‐weighted imaging may therefore offer a more sensitive, alternative method of imaging CALs.

Currently, the prevalence and temporal evolution of T_1_‐dark rims remain unknown, and their relation to PRLs requires further validation. The present study considers a dataset obtained by frequent 7 Tesla (T) scanning of untreated patients with MS acquiring magnetization prepared 2 rapid gradient echo (MP2RAGE), a T_1_‐weighted sequence that is more resistant to magnetic field inhomogeneities [12], and quantitative susceptibility mapping (QSM), which has better pathological sensitivity and specificity to iron than conventional SWI or phase maps [13]. Using this dataset, we have been able to perform an exploratory, longitudinal study reporting the prevalence and evolution of T_1_‐dark rims and PRLs in nascent and established white matter lesions.

## 2 Methods

### 2.1 Participants and Image Acquisition

The study was granted approval by a local ethics committee (05/Q2404/44), and all subjects signed an informed consent form prior to study enrolment. We analyzed scans from an exploratory pilot study with frequent scanning of four untreated patients with MS considering disease‐modifying therapy [14].

Patients underwent MRI scanning six times over 36 weeks, except for one patient who had five visits. Imaging was performed on a 7T Philips Achieva scanner with a single‐transmit 32‐channel head coil (Nova Medical). The specific acquisition parameters for each sequence are listed in Table 1. B_1_ maps were used to correct data for inevitable field variations within the scanner at 7T. We did not administer gadolinium contrast agent in this study.

**TABLE 1:** MRI sequence parameters.

Contrast	Resolution (mm^3^)	FOV (mm) (AP, RL, SI)	SENSE (RL, AP)	Readout BW (Hz)	TR (ms)	TE (ms)	TI (ms)	FA (°)	Duration (min)
FLAIR	0.6 × 0.6 × 0.6	192 × 180 × 120	1.5, 1	1599.0	4800	256	1650	90	5.28
T_1_w	0.6 × 0.6 × 0.6	200 × 180 × 120	2.2, 2	115.2	13	6	787	8	11.58
T_2_*w, QSM	0.6 × 0.6 × 0.6	192 × 185 × 70	2.5, 2	506.4	50	20		14	6.77
B_1_ map	3.2 × 4.0 × 4.0	205 × 180 × 132	No	4271.7	20, 120	5		60	3.05

Abbreviations: AP, anterior–posterior; BW, bandwidth; FA, flip angle; FLAIR, fluid‐attenuated inversion recovery; FOV, field of view; QSM, quantitative susceptibility mapping; RL, right–left; SENSE, sensitivity encoding; SI, superior–inferior; TE, echo time; TI, inversion time; TR, repetition time; w, weighted.

### 2.2 Image Postprocessing

MP2RAGE images were created using an in‐house‐developed approach [15]. T_2_*‐weighted fast field echo QSM data were reconstructed using STI Suite (v3.0, Berkeley, California, https://people.eecs.berkeley.edu/~chunlei.liu/software.html) [16]. A brain mask was created using the Brain Extraction Tool in FSL (v6.0.7.6., Oxford, UK, https://fsl.fmrib.ox.ac.uk/fsl/docs/#/) [17]. We used the Laplacian‐based phase unwrapping to process the signal phase, the V‐SHARP method with variable‐radius kernel at five voxels to reduce the sophisticated harmonic artifact for phase data, and the improved sparse linear equation and least‐square algorithm to generate susceptibility maps for each subject. QSM values were calculated relative to cerebrospinal fluid (CSF). This was achieved by averaging QSM values from four regions of interest in the CSF and subtracting that value from values found in other regions of interest. All images were rigidly registered to the baseline MP2RAGE scan using FSL FLIRT (Oxford, UK, https://fsl.fmrib.ox.ac.uk/fsl/docs/#/).

### 2.3 Rim Lesion Definitions and Analysis

T_1_‐dark rims were identified on MP2RAGE images according to the criteria used by Naval‐Baudin et al., which states that a hypointense ring of signal surrounding the lesion must be visible on two or more consecutive slices or on two orthogonal planes [8]. Any rims that did not fulfill the criteria (e.g., T_1_‐dark rim was clearly visible but only present on a single slice) were also recorded.

Paramagnetic rims manifesting as hyperintense rims on QSM were identified in line with the consensus definition [8]. A lesion with a paramagnetic rim can only be called a PRL if it does not enhance on a postcontrast scan or, in the absence of gadolinium contrast administration (as was the case in our study), the lesion's presence must be verified on a scan performed 3 months prior. To simplify terminology, we use the term “lesion with a QSM rim” and specify the number of possible PRLs (any lesions with a QSM rim) and true PRLs (lesions verified to be at least 3 months old with a QSM rim).

All the scans were reviewed by a single rater (M.M.) and subsequently reviewed by a senior researcher with 7 years of experience in PRL identification (M.A.C.). Any disagreements were discussed, and a consensus agreement was reached regarding the rim status of the lesion.

### 2.4 Cross‐Sectional Analysis of Pre‐Existing Lesions

Baseline white matter lesions visible on fluid‐attenuated inversion recovery (FLAIR) images were manually segmented, and their volumes were recorded using ITK‐SNAP (v.4.0.2, Philadelphia, PA, http://www.itksnap.org/pmwiki/pmwiki.php). The presence of a T_1_‐dark rim and/or QSM rim was recorded for each lesion.

### 2.5 Longitudinal Analysis of Pre‐Existing Lesions

The pre‐existing baseline lesions were reviewed at the final study visit to determine whether a T_1_‐dark rim and/or QSM rim were present. Only lesions with a QSM rim on the follow‐up scan (irrespective of QSM rim presence at baseline) were classed as true PRLs, in line with the consensus definition. Lesions with a QSM rim on the baseline scan only were classed as possible PRLs.

### 2.6 Longitudinal Analysis of New Lesions

Each scan was reviewed to identify new lesions that appeared during the study follow‐up (visits 2–6). These were segmented on both FLAIR and MP2RAGE images, and the volume of each lesion was calculated. All new lesions were then assessed for the presence of T_1_‐dark and QSM rims on all subsequent follow‐up scans. Only lesions with a QSM rim visible across three or more visits were classed as true PRLs.

Additionally, the detection of the T_1_‐dark rim was compared between the MP2RAGE and B_1_ maps to determine whether there was agreement between the two sequences. Regions with long T_1_ values appear hypointense on the MP2RAGE images and hyperintense on B_1_ maps.

Lastly, B_1_ maps were used to quantify longitudinal changes in T_1_ values in each new lesion. For all new lesions with a T_1_‐dark rim, the T_1_‐dark rim and lesion core were manually segmented. The masks were then applied to B_1_ maps, and mean T_1_ values were calculated for (1) new rimless lesions (based on the whole lesion mask), (2) the rim only of the lesions with a T_1_‐dark rim, and (3) the lesion core only of the lesions with a T_1_‐dark rim. This was done across all subsequent time points. Comparisons of the T_1_ values between the rimless and rim‐positive lesions were carried out within the same patients to account for possible intersubject and interscan differences.

### 2.7 Statistical Analysis

Considering the exploratory nature of the study and the small sample size, no formal statistical analyses were conducted. Descriptive statistics are provided.

## 3 Results

The baseline characteristics of the patients included in this study are presented in Table 2. The average interval between visits was 7.6 weeks, and the average total follow‐up period was 36 weeks.

**TABLE 2:** Patient characteristics.

Patient	Sex	Age	Disease subtype	Time since disease onset (months)	Number of lesions	Number of new lesions during the follow‐up
1	M	23	CIS	5	56	9
2	F	39	RRMS	261	22	1
3	F	35	CIS	1	32	2
4	F	27	RRMS	25	49	8

Abbreviations: CIS, clinically isolated syndrome; F, female; M, male; RRMS, relapsing‐remitting multiple sclerosis.

### 3.1 Cross‐Sectional Analysis

**FIGURE 1:**
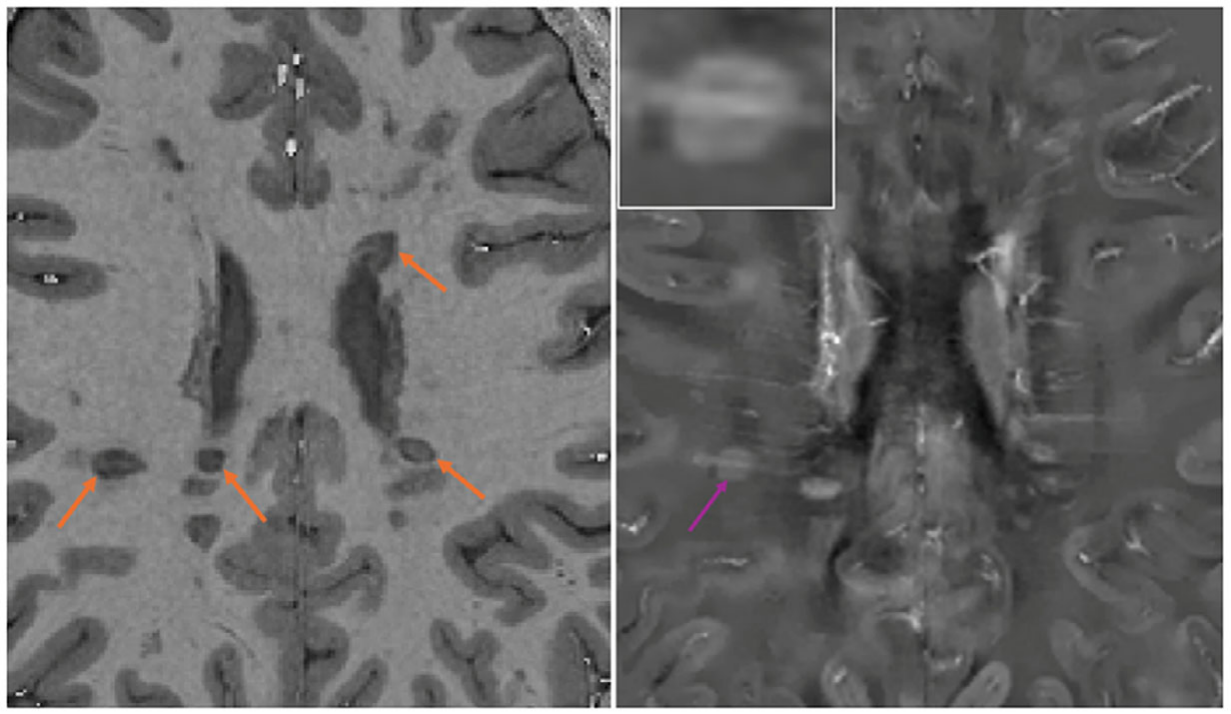
Lesions with T_1_‐dark rims on magnetization prepared 2 rapid gradient‐echo (marked by orange arrows) (left) and the corresponding quantitative susceptibility mapping (QSM) image showing a single paramagnetic rim lesion (marked by purple arrow) with a clear vein passing through its center (right). A magnification of the QSM‐rim is provided in the top left corner.

In total, 159 pre‐existing white matter lesions were identified on the baseline scans. A patient‐wise breakdown of all rim‐positive lesions is provided in Table 3. Examples of T_1_‐dark and QSM rims are shown in Figure 1.

**TABLE 3:** A summary of all rim‐positive lesions recorded at baseline and follow‐up.

	Number of rim‐positive lesions			
	Baseline		Final follow‐up (36 weeks later)	
Patient	T_1_‐dark	QSM	T_1_‐dark	QSM
1	19	8	14	7
2	1	0	1	0
3	2	2	2	2
4	0	1	2	2
Total lesions (%)	22 (13.8%)	11 (6.9%)	19 (11.9%)	11 (6.9%)

Abbreviation: QSM, quantitative susceptibility mapping.

#### 3.1.1 T_1_‐Dark Rims Are More Common Than QSM Rims

A total of 22 lesions (13.8%) were classed as having a T_1_‐dark rim and 11 (6.9%) as having a QSM rim. A further six lesions had a clear T_1_‐dark rim on one slice only and so were classified as rimless according to our protocol.

A total of 17 lesions had a T_1_‐dark rim only, six had a QSM rim only, and five lesions had both types of rims. Compared to the signal intensity of nearby white matter lesions, five of the six lesions with a QSM rim only appeared globally very hypointense on the MP2RAGE scans and one had patches of hypointense signal.

#### 3.1.2 Lesions With Rims Are Larger Than Rimless Lesions

**FIGURE 2:**
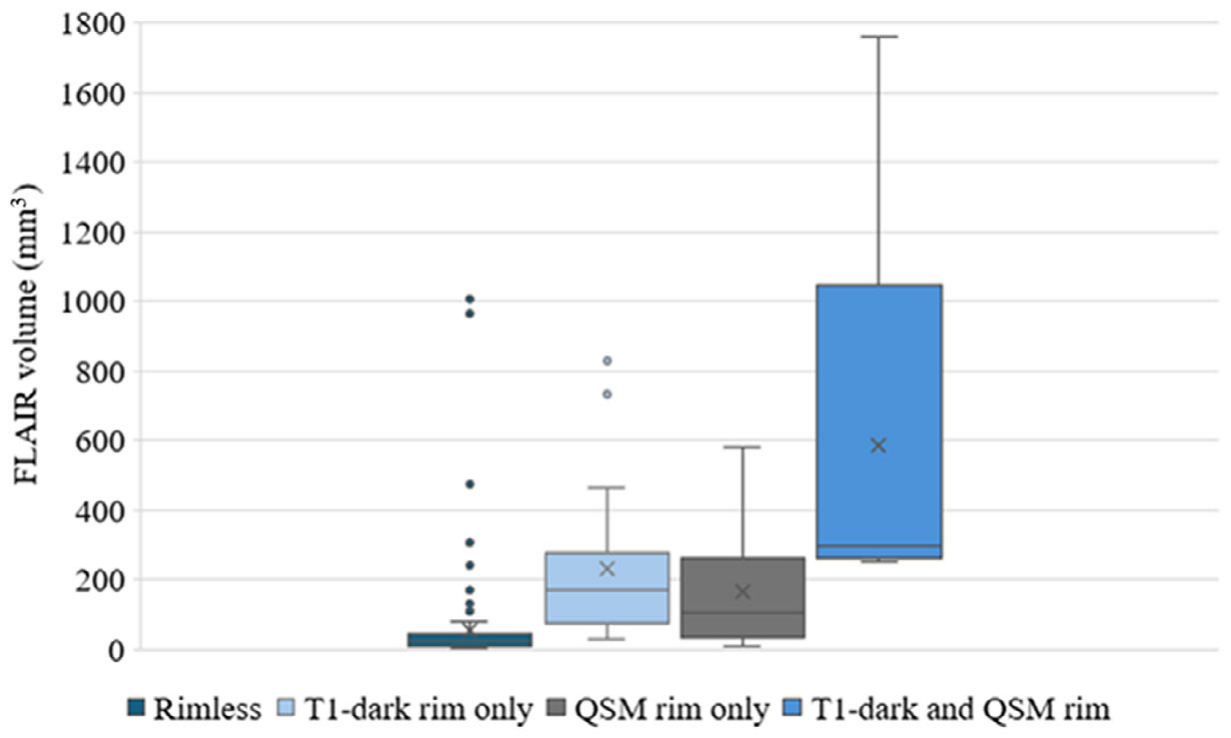
Baseline (pre‐existing) lesions with a T_1_‐dark rim only (*n* = 17), quantitative susceptibility mapping (QSM) rim only (*n* = 6), or both T_1_‐dark and QSM rims (*n* = 5) had a larger fluid‐attenuated inversion recovery (FLAIR) volume than lesions without either rims (*n* = 131). *n*, number of lesions.

Figure 2 shows the volumes of all lesions on the baseline scans according to rim status (T_1_‐dark, QSM, or both). The largest lesions had both T_1_‐dark and QSM rims, while most of the rimless lesions were noted to have a volume under 100 mm^3^.

### 3.2 Longitudinal Analysis of Pre‐Existing Lesions

A comparison of the total and patient‐wise numbers of pre‐existing lesions with T_1_‐dark and QSM rims over a short‐term follow‐up of 36 weeks is shown in Table 3.

#### 3.2.1 Lesions With T_1_‐Dark and QSM Rims Show Dynamic Changes on Short‐Term Follow‐Up

On the final follow‐up scan, a total of five pre‐existing lesions had both a T_1_‐dark and QSM rim. Two lesions had T_1_‐dark and QSM rims persisting from baseline to follow‐up. Three other lesions had a T_1_‐dark rim at baseline, but the QSM rim only appeared on the follow‐up scan. Four other T_1_‐dark rims developed during the study without QSM rims. At baseline, one of these appeared markedly hypointense on the MP2RAGE scan. Table 4 summarizes the temporal evolution of all pre‐existing lesions with a T_1‐_dark or QSM rim at the first and/or last study visit. Out of all the pre‐existing lesions studied, four were possible PRLs and 11 were true PRLs.

**TABLE 4:** Summary of the longitudinal evolution of lesions with T_1_‐dark rims and paramagnetic rim lesions between baseline and last follow‐up.

Rim lesion type	Number of rims persisting	Number of rims appearing	Number of rims disappearing
T_1_‐dark	14	5	8
QSM	7	4	4

Abbreviation: QSM, quantitative susceptibility mapping.

#### 3.2.2 T_1_‐Dark Rim Lesions Can Precede the Appearance of QSM Rims

Four pre‐existing lesions had a QSM rim appear at the final visit. Three of those QSM rims (75%) were preceded by a T_1_‐dark rim at baseline. The fourth lesion presented patches of hypointense signal in the lesion core on MP2RAGE at baseline. One baseline lesion with a QSM rim had a marked T_1_ hypointense signal throughout the lesion core and developed a T_1_‐dark rim at the follow‐up visit.

#### 3.2.3 T_1_‐Dark Rims Persist Longer Than QSM Rims

In two of the five (40%) pre‐existing lesions with both a T_1_‐dark rim and a QSM rim at baseline, the rims persisted 36 weeks later. For the remaining three lesions (60%), the T_1_‐dark rim persisted for the whole study period, but the QSM rim disappeared.

### 3.3 Longitudinal Analysis of New Lesions

**FIGURE 3:**
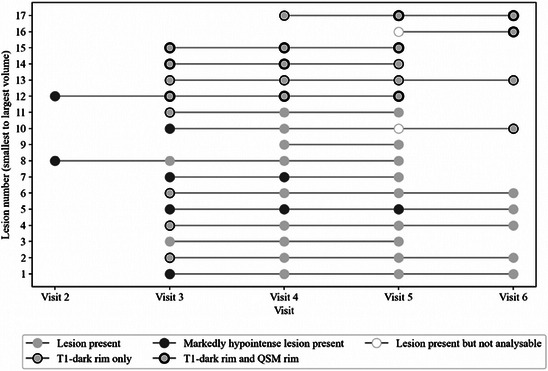
The temporal evolution of new lesions with T_1_‐dark and quantitative susceptibility mapping (QSM) rims. The lesions are arranged according to volume (from smallest to largest). Largest lesions had both T_1_‐dark and QSM rims, while smaller lesions had a transient T_1_‐dark rim or were markedly hypointense on the magnetization prepared 2 rapid gradient‐echo image. No new lesions had a QSM rim without also having a T_1_‐dark rim. Lesions not analyzable on QSM across the whole study duration were excluded. Two lesions were not analyzable at Visit 5 due to poor image quality. Lesions without data for visit 6 are all from Patient 4, who only had five visits.

A total of 20 new lesions appeared over the course of the study. Figure 3 describes the T_1_‐dark and QSM rim status of each new lesion.

#### 3.3.1 Most New Lesions Have a T_1_‐Dark Rim

Eleven lesions met the criteria for a T_1_‐dark rim, while a further five had a T_1_‐dark rim on one slice only and were classified as rimless. In eight of the 11 nascent lesions with a T_1_‐dark rim, the rim appeared at the first appearance of the lesion (see Figure 3). Five of the T_1_‐dark rims persisted for at least three visits and were present at the end of the data collection period. Another four T_1_‐dark rims disappeared after a single visit, while two lesions with T_1_‐dark rims only appeared on the final visit and consequently were not able to be followed up.

#### 3.3.2 New PRLs Are Accompanied by a T_1_‐Dark Rim

Five new lesions (25%) had a rim on QSM (see Figure 3). Four of these QSM rims appeared simultaneously to a T_1_‐dark rim, and the fifth QSM rim appeared one visit after the appearance of a T_1_‐dark rim. No new lesions had a QSM rim without also having a T_1_‐dark rim. Of the five new lesions with QSM and T_1_ rims, four had both rims still present at the final visit, while one lesion showed the QSM rim disappearing at the final visit with the T_1_ rim still present. Two of the five lesions with QSM rims had a rim visible across three visits and were therefore classed as true PRLs.

#### 3.3.3 The Presence of Rims Is Associated With the Size of the New Lesion

**FIGURE 4:**
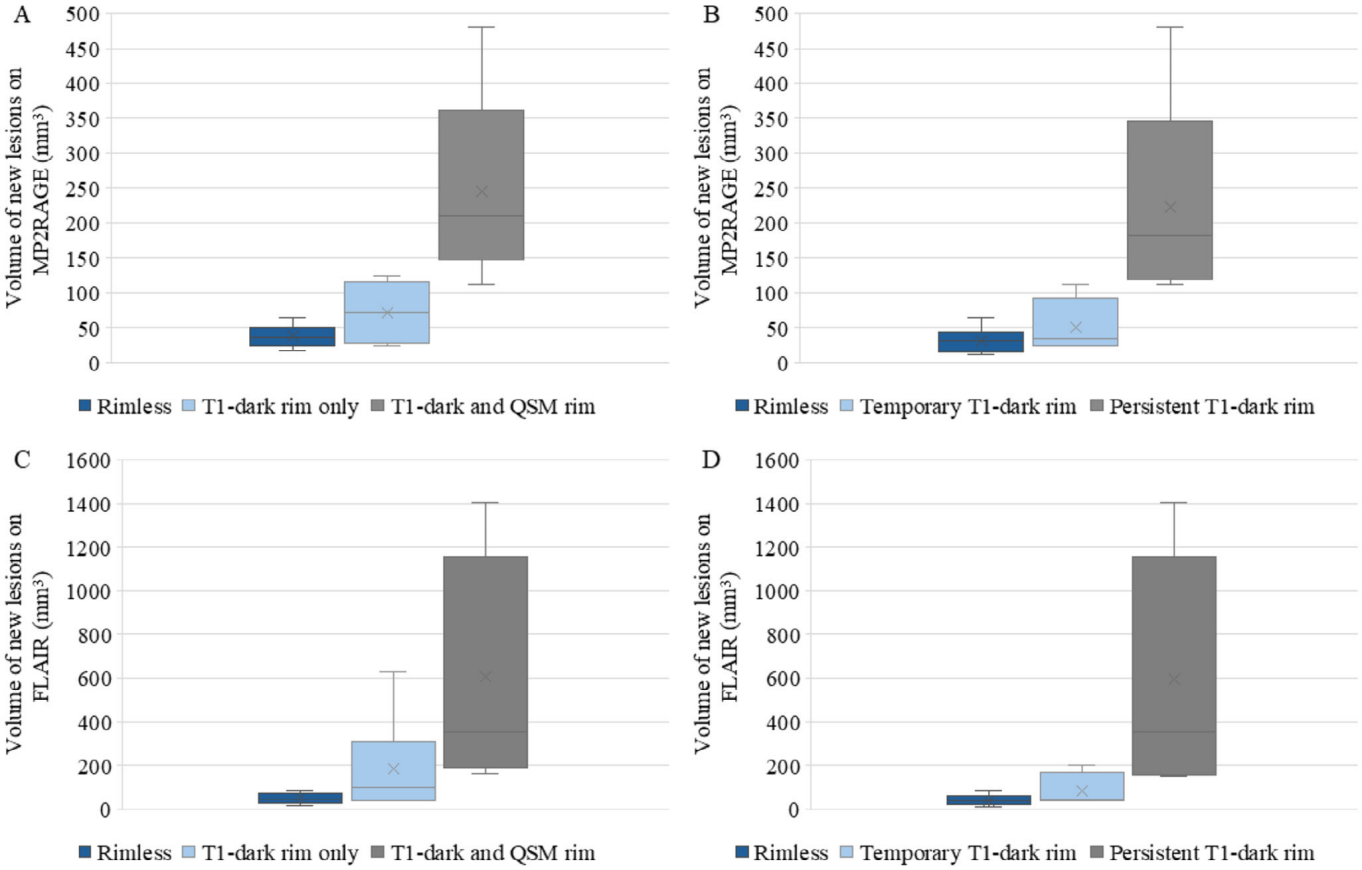
Magnetization prepared 2 rapid gradient‐echo (MP2RAGE) (top row) and fluid‐attenuated inversion recovery (FLAIR) (bottom row) volumes of new lesions according to rim status. (A) Average MP2RAGE volume of new rimless, T_1_‐dark rim only, and T_1_‐dark and quantitative susceptibility mapping (QSM) rim lesions. Three lesions without a T_1_ rim were unable to be assessed on QSM and were therefore excluded. (B) Average MP2RAGE volume of new lesions according to the temporal evolution of the T_1_‐dark rim: rimless, temporary T_1_‐dark rim, or persistent T_1_‐dark rim. Two lesions with rim appearing at the final visit were excluded. (C) Average FLAIR volume of new rimless, T_1_‐dark rim only, and T_1_‐dark and QSM rim lesions. (D) Average FLAIR volume of new lesions according to the temporal evolution of the T_1_‐dark rim: rimless, temporary T_1_‐dark rim, or persistent T_1_‐dark rim.

The presence of rims was related to the size of the lesions, as measured on FLAIR and MP2RAGE scans (see Figure 4). The largest lesions had both T_1_‐dark and QSM rims (see Figure 4A,C). Medium‐sized lesions had T_1_‐dark rims only. The smallest lesions were rimless. The persistence of T_1_‐dark rims was also related to the size of the lesion (see Figure 4B,D).

#### 3.3.4 MP2RAGE and B1 Maps Show High Agreement in Detecting T_1_‐Dark Rims

T_1_‐dark rims had consistently longer T_1_ values than the lesion core (data not shown). New lesions on B_1_ maps corresponded with what was observed on MP2RAGE in 18 of the 20 lesions (90%). One lesion only showed a rim on one slice on the MP2RAGE (and hence had been classed as rimless) but showed a rim on two slices on the B_1_ map. The other discrepancy was a large edematous lesion, which was classed as having a T_1_‐dark rim on MP2RAGE but was globally hyperintense on B_1_.

#### 3.3.5 The Core of New Rimless Lesions Is as Hypointense as the Rim of T_1_‐Dark Rim Lesions

**FIGURE 5:**
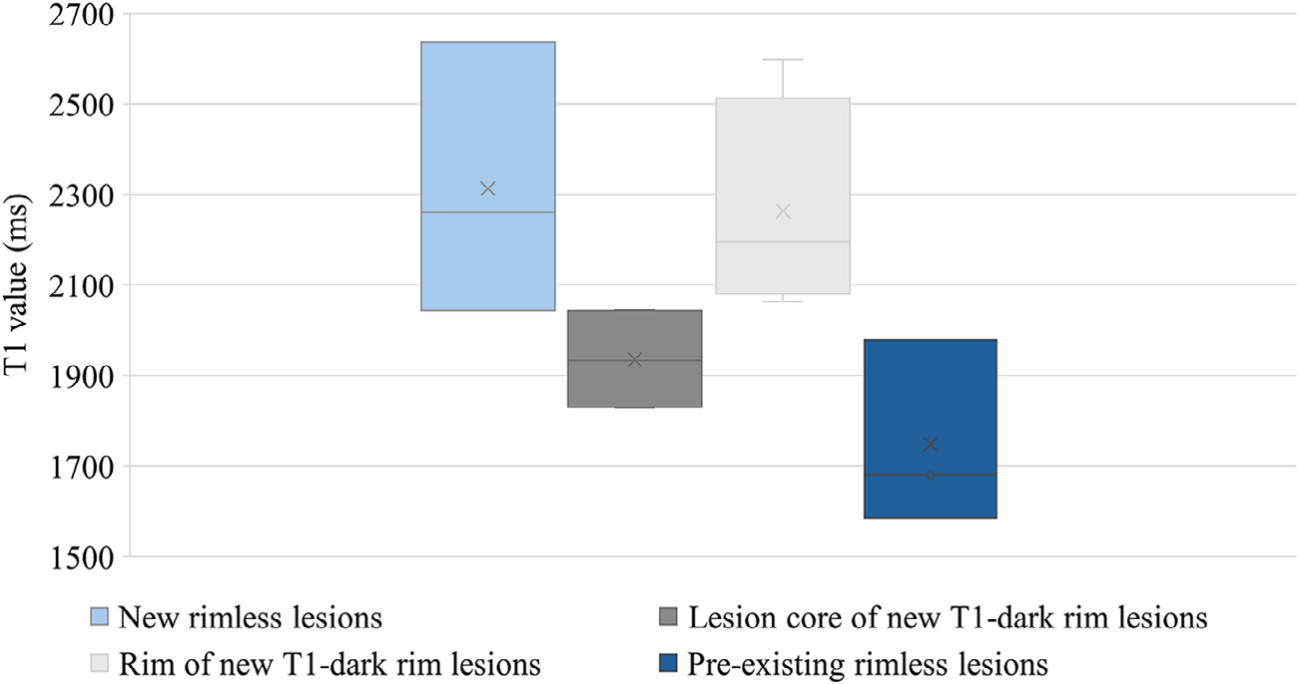
T_1_ values of new rimless lesions (whole lesion), lesion core and rim of new T_1_‐dark rim lesions (provided separately), and pre‐existing rimless lesions (whole lesion) for Patient 1. X shows mean values.

All the new lesions without a T_1_‐dark rim were small with a volume of less than 100 mm^3^ (see Figure 4). On MP2RAGE images, their core appeared diffusely hypointense with signal intensity similar to the rim rather than the core of the large, T_1_‐dark rim lesions. Calculations of T_1_ signal from B_1_ maps confirmed these findings. Moreover, new rimless lesions appeared to have higher T_1_ values when compared to similar‐sized, pre‐existing, rimless lesions. Example values from Patient 1, who had at least three lesions of each type, are shown in Figure 5. Consistent with pre‐existing small lesions having reduced T_1_ values, the T_1_ values of the small new lesions decreased over time (data not shown).

## 4 Discussion

This 7T MRI study presents novel findings on the prevalence and short‐term evolution of a newly described imaging marker, T_1_‐dark rims, and their relationship with PRLs detected on QSM. Our findings suggest that T_1_‐dark rims are sensitive at detecting, what we believe are, active MS lesions (either acute or chronic active).

We confirm observations from the first study to describe T_1_‐dark rims that they are more numerous than PRLs [11]. Using 7T imaging and QSM, we detected twice as many T_1_‐dark rims compared to PRLs, whereas in the 3T Naval‐Baudin et al. clinical study, T_1_‐dark rim lesions outnumbered PRLs by around a quarter. In addition to the MRI field strength, our increased T_1_‐dark rim detection rate is likely due to the differences in our methodologies; Naval‐Baudin and colleagues used SWI and phase imaging, which are not as pathologically specific to iron and, particularly phase, can lead to false positive findings [10]. In addition, by analyzing all brain lesions in a few patients with early MS, we were able to report the previously unknown prevalence of T_1_‐dark rims (14% of all lesions).

Our investigation of new lesions revealed a striking prevalence of T_1_‐dark rims. Over half of the new lesions displayed a T_1_‐dark rim, a proportion that rose to 80% when considering lesions exhibiting a rim on at least one slice. Our quantitative analyses confirmed visual observations that T_1_‐dark rims have longer T_1_ values than their lesion core. Several pathological processes associated with new lesion development can increase T_1_ signal, including demyelination, edema, phagocyte invasion, and hypercellularity [4, 18]. Based on the location of the signal, at the periphery of new lesions, we postulate that marked edema associated with acute inflammation, rather than demyelination, is the primary driver of the T_1_‐dark rim signal [19].

Notably, the remaining new lesions without a T_1_‐dark rim had small volumes, all under 100 mm^3^. Small lesions may lack visible rims due to their size (partial volume artifacts), and this could be tested by imaging at higher spatial resolution. However, comparing the T_1_ values of these small, new, rimless lesions to the rims and cores of larger T_1_‐dark rim‐positive lesions confirmed that their core T_1_ values matched the values observed at the T_1_‐dark rim, suggesting a shared pathological origin. Furthermore, small, new, rimless lesions had higher T_1_ values than size‐matched, rimless, older lesions, possibly indicating decreased inflammation and edema, consistent with pathological findings of lesions in the chronic inactive stage [20]. A subsequent T_1_ reduction over the follow‐up period likely reflects this progression.

T_1_‐dark rims were either transient (visible at only one visit) or persistent. A quarter of new lesions had transient T_1_‐dark rims, suggestive of subsiding edema. These lesions never developed a paramagnetic rim, suggesting a less aggressive disease pathology. Persistent T_1_‐dark rim lesions were always larger and accompanied by a paramagnetic rim, appearing either concurrently or one visit later. This indicates the presence of ongoing inflammation and tissue destruction, suggesting T_1_‐weighted imaging may offer a sensitive alternative for CAL detection, particularly as SWI is not currently recommended for MS monitoring.

While paramagnetic rims reflect iron‐laden macrophages and microglia, iron decreases T_1_ signal [21], opposing the increased T_1_ signal observed in T_1_‐dark rims. Thus, the T_1_‐dark rim is visible despite, not because of, iron accumulation. We speculate that paramagnetic rims may mark a more intense inflammatory and destructive process involving demyelination and macrophage‐mediated myelin (and iron) clearance, making them less common than inflammation alone at acute lesion borders. In another paper by Naval‐Baudin et al., the authors showed that deeply hypointense T_1_ voxels, often in the lesion periphery, were almost exclusively present in PRLs compared to non‐PRLs [22]. The association of both T_1_‐dark and paramagnetic rims with larger lesion sizes supports the hypothesis that both rim types are associated with a more aggressive disease pathology. These lesions may also represent a subset of slowly expanding lesions, a possibility requiring further investigation with longer follow‐up studies.

Future pathology and MRI studies will establish the role and long‐term evolution of T_1_‐dark rims in MS. For now, it is interesting to speculate whether the high incidence of T_1_‐dark rims in new lesions could potentially be used to provide evidence of dissemination in time (DIT) in patients presenting with suspected MS. Future studies could determine whether the simultaneous presence of rim‐positive and rim‐negative lesions can be used as evidence of DIT, similarly to the way gadolinium‐enhancing lesions are currently used. Moreover, T_1_‐dark rims could have a role in the monitoring of lesions or disease progression. Particularly, T_1_‐dark rims could act as surrogate markers of early and chronic inflammation and could therefore be used to assess the efficacy of novel disease‐modifying therapies such as Bruton's tyrosine kinase inhibitors, which act on both sides of the BBB. Given the existing, widespread use of T_1_‐weighted imaging in routine MS monitoring and the detection of T_1_‐dark rims by clinical 3T scanners [11], this new radiological sign could easily be implemented in clinical practice.

This analysis faces several limitations. First and foremost, our exploratory study included only four patients, and our results need to be validated in a larger sample size. Importantly, these data did not include contrast‐enhanced imaging, so it was not possible to definitively identify the acute stage of nascent lesions or determine a temporal correlation between T_1_‐dark rims and the resolution of gadolinium enhancement. However, it is known that MS lesions rarely enhance beyond 2–3 months [23], and T_1_‐dark rims were seen to persist for 5 months, until the end of the data collection period.

Furthermore, since nascent lesions with a persistent T_1_‐dark rim and paramagnetic rim were still observed at the end of the study, interpretation of the long‐term evolution of T_1_‐dark rims and the temporal correlation with paramagnetic rims is limited. Further longitudinal follow‐up and inclusion of imaging techniques such as proton density, to confirm whether T_1_‐dark rims represent edema, and diffusion‐weighted imaging, to infer the underlying tissue integrity, will be useful in discerning the underlying pathology of T_1_‐dark rims and differences between them and PRLs.

This study further characterized the novel imaging finding of T_1_‐dark rims that surround some MS lesions. We propose that T_1_‐dark rims may represent a marker of early lesions with ongoing edema/inflammation at the edges surrounding demyelination. Considering that T_1_‐weighted imaging is one of the most commonly utilized sequences, T_1_‐dark rims could easily be incorporated into routine, clinical assessment of patients with MS. However, future large, longitudinal studies are needed to further establish their role, evolution, and underlying pathology.

## Conflicts of Interest

The authors declare no conflicts of interest

## References

[1] C. W. M. Adams , R. N. Poston , and S. J. Buk , “Pathology, Histochemistry and Immunocytochemistry of Lesions in Acute Multiple Sclerosis,” Journal of the Neurological Sciences 92, no. 2–3 (1989): 291–306. 2809622 10.1016/0022-510x(89)90144-5

[2] M. H. Barnett and J. W. Prineas , “Relapsing and Remitting Multiple Sclerosis: Pathology of the Newly Forming Lesion,” Annals of Neurology 55, no. 4 (2004): 458–468, 10.1002/ana.20016. 15048884

[3] A Hirano , “Edema and Myelin‐Associated Extracellular Spaces,” in Brain Edema, ed. Y. Inaba , I. Klatzo , and M. Spatz (Springer, 1985), 6–13.

[4] M. Filippi , M. A. Rocca , F. Barkhof , et al., “Association Between Pathological and MRI Findings in Multiple Sclerosis,” Lancet Neurology 11, no. 4 (2012): 349–360, https://www.sciencedirect.com/science/article/pii/S1474442212700030. 22441196 10.1016/S1474-4422(12)70003-0

[5] M. I. Gaitán , C. D. Shea , I. E. Evangelou , et al., “Evolution of the Blood‐Brain Barrier in Newly Forming Multiple Sclerosis Lesions,” Annals of Neurology 70, no. 1 (2011): 22–29. 21710622 10.1002/ana.22472 PMC3143223

[6] M. Absinta , P. Sati , M. I. Gaitán , et al., “Seven‐Tesla Phase Imaging of Acute Multiple Sclerosis Lesions: A New Window Into the Inflammatory Process,” Annals of Neurology 74, no. 5 (2013): 669–678. 23813441 10.1002/ana.23959 PMC3812397

[7] J. W. Prineas , E. E. Kwon , E. S. Cho , et al., “Immunopathology of Secondary‐Progressive Multiple Sclerosis,” Annals of Neurology 50, no. 5 (2001): 646–657. 11706971 10.1002/ana.1255

[8] F. Bagnato , P. Sati , C. C. Hemond , et al., “Imaging Chronic Active Lesions in Multiple Sclerosis: A Consensus Statement,” Brain 147, no. 9 (2024): 2913–2933. 38226694 10.1093/brain/awae013 PMC11370808

[9] P. Preziosa , M. Filippi , and M. A. Rocca , “Chronic Active Lesions: A New MRI Biomarker to Monitor Treatment Effect in Multiple Sclerosis?,” Expert Review of Neurotherapeutics 21, no. 8 (2021): 837–841, 10.1080/14737175.2021.1953983. 34236010

[10] A. Dal‐Bianco , J. Oh , P. Sati , and M. Absinta , “Chronic Active Lesions in Multiple Sclerosis: Classification, Terminology, and Clinical Significance,” Therapeutic Advances in Neurological Disorders 17 (2024), 10.1177/17562864241306684. PMC11660293 39711984

[11] P. Naval‐Baudin , A. Pons‐Escoda , A. Castillo‐Pinar , et al., “The T1‐Dark‐Rim: A Novel Imaging Sign for Detecting Smoldering Inflammation in Multiple Sclerosis,” European Journal of Radiology 173 (2024): 111358, https://www.sciencedirect.com/science/article/pii/S0720048. 38340569 10.1016/j.ejrad.2024.111358

[12] J. P. Marques , T. Kober , G. Krueger , W. van der Zwaag , P. F. Van de Moortele , and R. Gruetter , “MP2RAGE, a Self Bias‐Field Corrected Sequence for Improved Segmentation and T1‐Mapping at High Field,” Neuroimage 49, no. 2 (2010): 1271–1281. 19819338 10.1016/j.neuroimage.2009.10.002

[13] K. M. Gillen , T. D. Nguyen , A. Dimov , et al., “Quantitative Susceptibility Mapping Is More Sensitive and Specific Than Phase Imaging in Detecting Chronic Active Multiple Sclerosis Lesion Rims: Pathological Validation,” Brain Communications 7 (2025): fcaf011. 39916751 10.1093/braincomms/fcaf011 PMC11800486

[14] K. Aphiwatthanasumet , O. Mougin , N. Geades , et al., “A Longitudinal Study of Lesion Evolution in Multiple Sclerosis Using Multi‐Contrast 7T MRI,” paper presented at the 26th Annual Meeting of the International Society for Magnetic Resonance in Medicine, Paris, France, June 16–21, 2018.

[15] O. Mougin , R. Abdel‐Fahim , R. Dineen , A. Pitiot , N. Evangelou , and P. Gowland , “Imaging Gray Matter With Concomitant Null Point Imaging From the Phase Sensitive Inversion Recovery Sequence,” Magnetic Resonance in Medicine 76, no. 5 (2016): 1512–1516, 10.1002/mrm.26061. 26599705 PMC5082579

[16] W. Li , A. V. Avram , B. Wu , X. Xiao , and C. Liu , “Integrated Laplacian‐Based Phase Unwrapping and Background Phase Removal for Quantitative Susceptibility Mapping,” NMR in Biomedicine 27, no. 2 (2014): 219–227. 24357120 10.1002/nbm.3056 PMC3947438

[17] M. Jenkinson , C. F. Beckmann , T. E. J. Behrens , M. W. Woolrich , and S. M. Smith , “FSL,” Neuroimage 62, no. 2 (2012): 782–790. 21979382 10.1016/j.neuroimage.2011.09.015

[18] M. A. A. van Walderveen , W. Kamphorst , P. Scheltens , et al., “Histopathologic Correlate of Hypointense Lesions on T1‐Weighted Spin‐Echo MRI in Multiple Sclerosis,” Neurology 50, no. 5 (1998): 1282–1288, 10.1212/WNL.50.5.1282. 9595975

[19] F. Barkhof and K. K Koeller , “Demyelinating Diseases of the CNS (Brain and Spine),” in Diseases of the Brain, Head and Neck, Spine 2024–2027: Diagnostic Imaging, ed. J. Hodler , R. A. Kubik‐Huch , and J. E. Roos (Springer, 2024), 189–202, 10.1007/978-3-031-50675-8_13. 39495887

[20] H. Lassmann , J. van Horssen , and D Mahad , “Progressive Multiple Sclerosis: Pathology and Pathogenesis,” Nature Reviews Neurology 8, no. 11 (2012): 647–656, 10.1038/nrneurol.2012.168. 23007702

[21] T. Kanda , Y. Nakai , S. Aoki , et al., “Contribution of Metals to Brain MR Signal Intensity: Review Articles,” Japanese Journal of Radiology 34, no. 4 (2016): 258–266, 10.1007/s11604-016-0532-8. 26932404

[22] P. Naval‐Baudin , A. Pons‐Escoda , À. Camins , et al., “Deeply 3D‐T1‐TFE Hypointense Voxels Are Characteristic of Phase‐Rim Lesions in Multiple Sclerosis,” European Radiology 34, no. 2 (2024): 1337–1345. 37278854 10.1007/s00330-023-09784-w PMC10853299

[23] À. Rovira , F. M. Doniselli , C. Auger , et al., “Use of Gadolinium‐Based Contrast Agents in Multiple Sclerosis: A Review by the ESMRMB‐GREC and ESNR Multiple Sclerosis Working Group,” European Radiology 34, no. 3 (2024): 1726–1735, 10.1007/s00330-023-10151-y. 37658891

